# Contribution of methamphetamine and insulin to the death of a woman suffering from type I diabetes – which played the greater role?

**DOI:** 10.1007/s12024-024-00855-y

**Published:** 2024-07-04

**Authors:** Klára Handlosová, Pavel Šištík, Matěj Uvíra, Kateřina Andelova, Petr Handlos, David Stejskal

**Affiliations:** 1 Department of Forensic Medicine, University Hospital Ostrava, Ostrava, 70852 Czech Republic; 2 Department of Forensic Medicine, Faculty of Medicine, University of Ostrava, Ostrava, 701 03 Czech Republic; 3 Department of Clinical Pharmacology, Institute of Laboratory Medicine, University Hospital Ostrava, 17. listopadu 1790, Ostrava, 708 52 Czech Republic; 4 Institute of Laboratory Medicine, Faculty of Medicine, University of Ostrava, Syllabova 19, Ostrava, 703 00 Czech Republic; 5 Department of Clinical Pharmacology, Faculty of Medicine, University of Ostrava, Syllabova 19, Ostrava, 703 00 Czech Republic; 6 Department of Clinical Biochemistry, Institute of Laboratory Medicine, University Hospital Ostrava, Ostrava, Czechia

**Keywords:** Type I diabetes, Methamphetamine, Insulin overdose, Hypoglycemia, Legal consequences

## Abstract

This report presents a fatal case of a young female Type I diabetic patient who developed convulsions and loss of consciousness after taking methamphetamine and spending some time in a dance club. During the convulsions, she was given sugar and when no response occurred, her boyfriend who was not experienced in the use of insulin administered a dose of insulin to her. The woman lost consciousness and died despite the efforts of the emergency service. A biochemical analysis revealed a high level of insulin (196.67 mU/L) and low levels of glucose (2.96 mmol/L) and C-peptide (26 pmol/L). Toxicological analysis revealed a methamphetamine concentration of 389 ng/mL and an amphetamine concentration of 19 ng/mL. The forensic perspective of the difficult determination of the contribution of each of the factors to the death, i.e., the pre-existing medical condition (Type I diabetes), the use of methamphetamine, the physical exertion at the dance club, and, finally, the non-indicated administration of insulin, is discussed. The ruling of the court is also reported.

## Introduction

The presented case describes the forensic and subsequent legal aspects of a fatal case of combined methamphetamine and insulin intoxication leading to death in a woman with Type I diabetes. These two substances were applied within a short period before death. In the Czech Republic, a forensic autopsy is ordered in all cases in which the law enforcement authorities take suspicion that the death resulted from a crime [1]. The forensic autopsy always includes two forensic medicine experts’ reports [2]. If the involvement of foreign substances is suspected, additional laboratory tests must be carried out and a toxicologist’s expert opinion is required. The expert reports must then be defended by the experts at the court and the court decides on the guilt or innocence and, if applicable, on the sentence.

## Case report

In the early morning hours, the emergency rescue center received a phone call reporting the collapse of a 27-year-old Type I diabetic female. According to the initial information, the woman had spent the evening at a dance club; upon her return home, she developed tonic-clonic convulsions and unconsciousness requiring cardiopulmonary resuscitation. Upon the arrival of the ambulance, the doctor and paramedic continued in extended resuscitation. The patient was placed on mechanical ventilation and, due to pulseless ventricular tachycardia, defibrillation was performed. The glucose levels were below the glucometer detection limit (< 1 mmol/L). During the resuscitation, the patient received adrenalin and a 40% glucose solution with no effect. After 30 min, resuscitation was terminated for futility. The medical records showed that the patient suffered from insulin-dependent Type I diabetes (10 units of Novorapid insulin three times a day, 10 units of Lantus at night).

### Autopsy and histological findings

An autopsy was performed 20 h after the death. An external examination revealed only insignificant superficial traumatic changes on the body of the deceased including skin abrasions and hematomas. An injection mark covered with medical tape was found in the left cubital fossa as a result of the administration of the glucose solution by the emergency doctor. Punctate skin defects with a reddish base were found in the right thigh and in the left cubital fossa, probably injection marks of older date.

Internal examination revealed cerebral and pulmonary edema. The lungs showed reduced aeration. The tissue was stiff, leaking frothy, blood-colored fluid, which was also present in the airways. The brain was enlarged (1350 g) with well-developed signs of edema. Ecchymoses were noted on the serosal membranes, all organs were congested. Liquid blood was present in the large vessels. It should be, however, noted that the signs of pulmonary and brain edema could have been emphasized by the resuscitation.

The autopsy also revealed the presence of diseases such as slight fibrosis of the mitral valve and the intracardiac membrane in the left atrium and small hepatic hemangioma.

Histological examination showed severe pulmonary and brain edema with well-developed distention of perivascular and pericellular spaces. Microscopic examination also ruled out any signs of known serious acute complications associated with amphetamine use (myocardial infarction, cerebral hemorrhage). All examined organs were congested.

### Postmortem biochemistry and toxicology

Insulin and C-peptide were measured using a chemiluminescent method on an Atellica Solution IM 1300 analyzer (Siemens Healthineers, USA). Glucose and lactate concentrations were measured spectrophotometrically using an Atellica Solution CH 930 analyzer (Siemens Healthineers, USA). To reduce the effect of hemolysis interference, the sample was diluted 10 times. A biochemical examination of the blood sample taken at the autopsy revealed a high level of insulin, consistent with its external administration (196.67 mU/L, normal range 3.00–25.00 mU/L) and a low level of glucose (2.96 mmol/L, normal range 3.60–5.59 mmol/L). A low level of glucose was also found in the vitreous body (1.59 mmol/L). In addition, a low level of C-peptide was found (26 pmol/L, normal range 268–1274 pmol/L).

Methamphetamine and amphetamine were analyzed by liquid chromatography–mass spectrometry (Agilent Technologies, USA). Toxicological analysis of the blood sample revealed a methamphetamine concentration of 389 ng/mL and an amphetamine concentration of 19 ng/mL. In addition, hair was analyzed for the presence of amphetamines. A 5 cm long strand of hair was taken for analysis and cut into three equal-length segments. The methamphetamine at concentrations of 1.21–1.51 ng/mg was detected in all hair segments, indicating that the victim was a chronic user of methamphetamine. The alcohol test was negative.

### Cause of death

The immediate cause of death of the deceased with insulin-dependent Type I diabetes was hypoglycemia caused by the effects of methamphetamine and insulin.

### Legal consequences

**Fig. 1:**
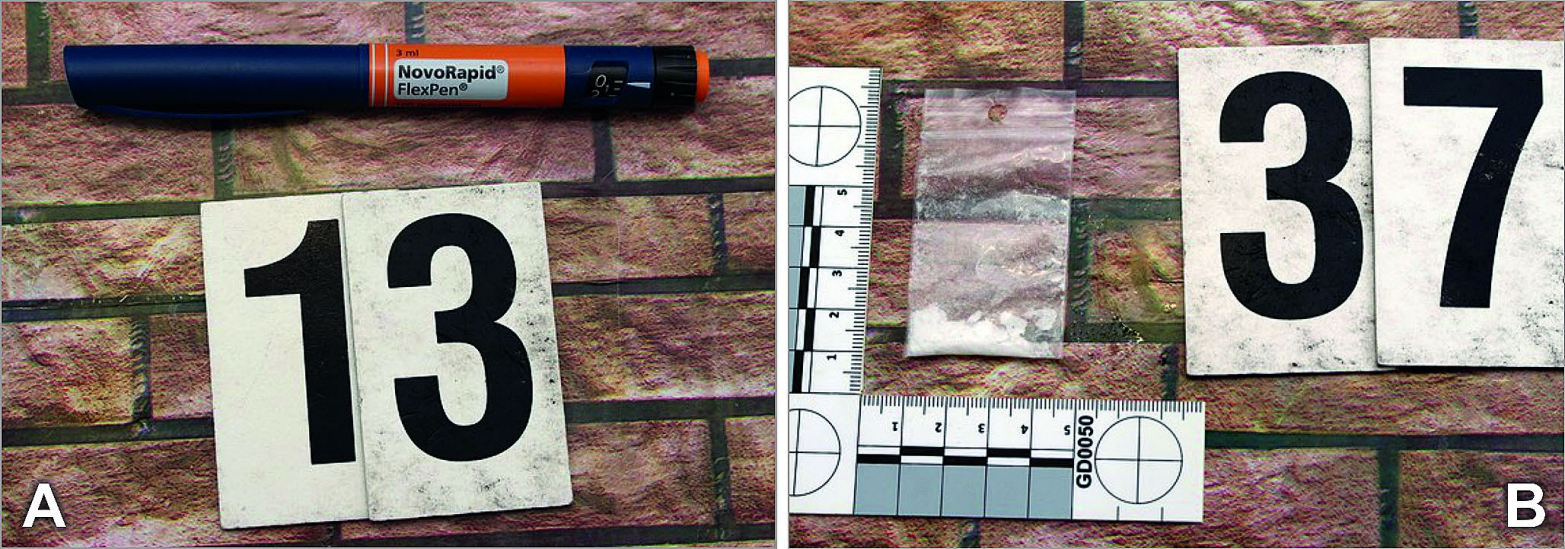
(**A**) Novorapid insulin pen which was used to administer insulin to victim. Insulin pen was collected at the crime scene. (**B**) Plastic bag containing methamphetamine found and collected during body inspection of one of the men

The police investigation revealed that on the day in question, the woman’s partner and two other young men were in the apartment with the deceased. After being presented with the autopsy report, they told the police that the victim had intranasally used approximately 0.1 g of methamphetamine before going to the club in the evening. The drug had been provided by one of the men. After returning from the club, the victim intranasally used another 0.2 g of methamphetamine. Approximately two or three hours later, the woman began to collapse and developed tonic-clonic convulsions. The woman’s partner put a spoonful of sugar in the woman’s mouth during the convulsions. As the convulsions continued, he put a square of chocolate in her mouth approximately 1 min later. After about another minute, he injected the woman with 6 units of the Novorapid insulin using her insulin pen (Fig. 1A). When asked by the investigator whether he had measured her blood glucose level before administering the insulin, he replied that he had not, that he did not know how to do it and had never done it before. According to the partner, the woman calmed down after the administration of insulin, breathed deeply, started to sweat, and, shortly afterward, lost consciousness. The partner then called an ambulance. The body inspection of one of the men performed by the police revealed a plastic bag containing methamphetamine (Fig. 1B).

## Discussion

Determining the cause of death in diabetics is often challenging, especially due to the need to perform an autopsy within a short time after death due to the rapid degradation of relevant biological markers (insulin, glucose, C-peptide, etc.) [3, 4].

Diabetic patients may be at imminent risk of death if acute complications of diabetes (hypoglycemia or hyperglycemia) occur [5, 6]. Hyperglycemia is characterized by glycosuria, osmotic diuresis, and severe dehydration of the body, all of which can eventually lead to the disruption of the internal environment and to impaired consciousness [7]. Type I diabetics are also at risk of the accumulation of ketones formed in the liver, which can lead to ketoacidotic coma [8, 9].

In clinical practice, hypoglycemia (blood glucose falling below 3.9 mmol/L) is a frequently encountered complication in Type I diabetics on insulin therapy. It is usually caused by an inadequate insulin dose, excessive physical activity, or reduced food intake [10, 11].

In the presented case, a woman with Type I diabetes used methamphetamine as documented by both the witness testimonies and the results of the toxicological analysis. Methamphetamine is known for its stimulant effects, manifested by a feeling of a significant increase in energy, relaxation, and euphoria, which are accompanied by increased glucose consumption. In addition, methamphetamine is known to suppress appetite, which can also lead to the development of hypoglycemia in patients with Type I diabetes [12]. It has to be noted that in individuals with no pre-existing medical condition (such as Type I diabetes), the concentration of methamphetamine detected in the blood of the deceased woman is not usually life-threatening [13]. Furthermore, the autopsy and subsequent histological examination did not reveal any signs of known serious acute complications associated with methamphetamine and amphetamine use (myocardial infarction, cerebral hemorrhage) [14]. On the other hand, long-term users may develop homeostasis disruption that can potentially lead to death [15].

In addition to general congestion of the organism, lung and brain edemas were found during the autopsy. These signs may have developed as a result of the detected hypoglycemia, but they might have also resulted from the resuscitation. The same is to be said for the frothy, bloody content of the airways and the congestion of the lungs. These findings are common after unsuccessful resuscitation.

In the presented case, the development of convulsions and collapse could have been provoked by hypoglycemia as a result of Type I diabetes, hypoglycemia due to methamphetamine abuse, but also due to a combination of both these factors [10, 16]. After the development of convulsions, the woman was given insulin which was not indicated as evidenced by the results of the biochemical analysis. In this case, it is reasonable to assume that the fatal homeostasis disruption was caused by hypoglycemia induced by a combination of all these factors. Unfortunately, forensic practice cannot provide a definitive answer to the question of whether any of these factors alone would be capable of causing death in this case, nor can it quantify the contribution of each to the fatal outcome.

The court and the prosecution were then faced with the difficult task of determining the role of the two men in the death of the young woman and their guilt. In the Czech Republic, crimes of negligence are tried in district courts, while criminal acts involving death due to the influence of drugs are tried in regional (higher degree) courts. In effect, each of the defendants was tried in a different court with a different panel of judges. The man who provided the woman with methamphetamine was charged by the prosecutor with the offense of illicit production and dealing with narcotic drugs, psychotropic substances, and poisons, which is punishable by 10–18 years of imprisonment [1]. The first instance court ruled that the direct causal link between the administration of methamphetamine and death could not be proved beyond a reasonable doubt and sentenced the defendant to 5 years in a maximum-security prison for the distribution of an addictive substance. The appellate court, however, ruled that the causal link existed, even though it could not have been quantified, and convicted the defendant of causing death by providing an illicit narcotic substance, sentencing him to 11 years in a maximum-security prison.

The partner of the deceased woman who administered insulin was charged with manslaughter by negligence, with a penalty of up to three years. In his case, the court requested an additional expert opinion from the field of internal medicine for a specific statement on the possible role of insulin in the case. The expert’s report concluded that the amount of insulin mentioned in the witness statement would not endanger the life of a well-treated diabetic. The amount of the actually administered insulin could, however, not have been verified. The expert was unable to comment with certainty on whether the woman’s life could have been saved at the time when her convulsions started if she had received no insulin. Although it is clear that the administration of insulin to hypoglycemic patients aggravates hypoglycemia, thereby reducing the possible chances of rescue, the court had to take into account the fact that neither the post-mortem laboratory examination nor any other expert examination could prove beyond reasonable doubt that the woman died as a result of hypoglycemia aggravated by the administration of insulin and, therefore, the court relied on the principle of in dubio pro reo. For these reasons, the defendant was eventually acquitted of all charges, even though he had no expertise or training to handle and administer drugs to another person.

In conclusion, we presented a case of a young female diabetic patient on insulin therapy whose death resulted from a combination of her illness, the use of methamphetamine, and insulin. In forensic practice, it is very difficult (sometimes even impossible) to quantify the contribution of individual factors to death in similar cases. Such equivocal conclusions, when a direct link between the administration of a foreign substance and the death cannot be proven, put the law enforcement authorities in a difficult position.

## Key points

1) Death of a Type I diabetic patient after the use of methamphetamine.

2) Insulin misadministration to a Type I diabetic patient.

3) Detailed forensic evaluation of the case.

4) Challenges in the determination of the contribution of each factor to the death are discussed.

## Declarations

### Article classifications

10: Forensics / 10.010: Pathology, 10.020: Medicine, 10.190: Autopsy.
